# Exploring the Prevalence of Antimicrobial Resistance in *Salmonella* and commensal *Escherichia coli* from Non-Traditional Companion Animals: A Pilot Study

**DOI:** 10.3390/life14020170

**Published:** 2024-01-24

**Authors:** Ana Marco-Fuertes, Santiago Vega, José Villora-Gonzalez, Clara Marin, Laura Montoro-Dasi

**Affiliations:** 1 Departamento de Producción y Sanidad Animal, Salud Pública Veterinaria y Ciencia y Tecnología de los Alimentos, Facultad de Veterinaria, Instituto de Ciencias Biomédicas, Universidad Cardenal Herrera-CEU, CEU Universities, Calle Santiago Ramón y Cajal 20, 46115 Alfara del Patriarca, Valencia, Spain; ana.marcofuertes@uchceu.es (A.M.-F.); svega@uchceu.es (S.V.); laura.montoro@uchceu.es (L.M.-D.); 2 Selvätica Veterinary Clinic, 46020 Valencia, Spain; josevilloragon@gmail.com

**Keywords:** antimicrobial resistance, *Escherichia coli*, multi-drug resistance, non-traditional companion animals, *Salmonella*, small mammals, zoonoses

## Abstract

Companion animal ownership has evolved to new exotic animals, including small mammals, posing a new public health challenge, especially due to the ability of some of these new species to harbour zoonotic bacteria, such as *Salmonella*, and spread their antimicrobial resistances (AMR) to other bacteria through the environment they share. Therefore, the objective of the present pilot study was to evaluate the current epidemiological AMR situation in commensal *Escherichia coli* and *Salmonella* spp., in non-traditional companion animal small mammals in the Valencia region. For this purpose, 72 rectal swabs of nine different species of small mammals were taken to assess the antimicrobial susceptibility against 28 antibiotics. A total of one *Salmonella enterica* serovar Telelkebir 13,23:d:e,n,z_15_ and twenty commensal *E. coli* strains were isolated. For *E. coli* strains, a high prevalence of AMR (85%) and MDR (82.6%) was observed, although neither of them had access outside the household. The highest AMR were observed in quinolones, one of the highest priority critically important antimicrobials (HPCIAs) in human medicine. However, no AMR were found for *Salmonella*. In conclusion, the results showed that small mammals’ commensal *E. coli* poses a public health risk due to the high AMR found, and the ability of this bacterium to transmit its resistance genes to other bacteria. For this reason, this pilot study highlighted the need to establish programmes to control AMR trends in the growing population of new companion animals, as they could disseminate AMR to humans and animals through their shared environment.

## 1. Introduction

Traditionally, only dogs and cats have been considered companion animals due to their close relationship with humans, either because they have been used as working animals or because they share a household [1]. Today, however, trends in pet ownership are evolving towards exotic animals such as snakes, lizards, exotic birds, rabbits or ferrets, among others, whose populations have increased by almost 25% in the last decade [2,3]. These animals are classified as non-native species in their current habitat, encompassing new species that are legally allowed to be kept at home [4]. However, the British Small Animal Veterinary Association (BSAVA) refers to them as non-traditional companion animals (NTCAs), as this term better describes the species in question. This is because some species may be indigenous to one country rather than another yet have recently been introduced into households as companion animals [4]. Several research studies highlight the importance of NTCAs, such as reptiles, as they can act as reservoirs and sources of *Salmonella* infection [5,6], a bacterium that is recognised by the World Health Organisation (WHO) as one of the priority pathogens with higher antimicrobial resistance (AMR) worldwide, which needs to be studied and monitored [7]. In fact, numerous outbreaks have occurred in recent years due to the close contact between these animals and their owners, causing serious disease or even death in at-risk populations such as immunocompromised patients, children or the elderly [5,8]. However, little is known about the importance of this pathogen in NTCA small mammals, even though the population of these animals, such as rabbits, guinea pigs, ferrets or mice, among others, is estimated at 29 million of the 340 million companion animals in European households [2].

This growing population has raised new public health concerns, especially in the transmission of AMR genes, as these companion animals are increasingly in close contact with their owners. Therefore, the rise of antimicrobial-resistant bacteria in both animals and humans poses a significant global risk to public health, directly related to the likelihood of antibiotic therapy failure. In fact, according to the WHO, AMR and the appearance of multidrug resistance (MDR) are one of the most important problems faced by public health worldwide [9].

In this line, the European Food and Safety Authority (EFSA) has routinely monitored AMR in commensal bacteria, such as *E. coli* [10], and zoonotic pathogens, such as *Salmonella* [11]. Traditionally, the focus has been on monitoring their transmission from food-producing animals to humans, as the role of companion animals was not considered a significant risk for AMR transmission and, therefore, was not included in these surveillance programmes. However, due to the rise of AMR and the emergence of MDR, it is necessary to analyse potential sources of AMR, and therefore, to expand these programmes to encompass a broader range of animal species. Thus, the European Union (EU) is developing the European Antimicrobial Resistance Surveillance Network in Veterinary Medicine (EARS-Vet) [12], a programme in veterinary medicine that harmonises the current ones and includes dogs and cats. This programme aims to complement the one already implemented in human medicine, the European Antimicrobial Resistance Surveillance Network (EARS-Net) [13], to underpin this problem under the One Health strategy to develop collaboration between human, veterinary and environmental health [9]. 

However, NTCAs are still not included in monitoring and surveillance programmes, resulting in a lack of information addressing the epidemiologic situation of AMR in small mammals as new companion animals, and the potential for transmission of AMR genes and zoonotic pathogens to humans through the home environment they share [14,15]. Therefore, the objective of this pilot study was to assess the prevalence of commensal *E. coli* and whether the study population carried *Salmonella*, along with its AMR and MDR patterns, in small mammals from the Valencia region.

## 2. Materials and Methods

### 2.1. Experimental Design

The present study was conducted in Valencia region. To this end, a large-scale veterinary centre (VC), serving almost 70% of the exotic animals in the Valencia region, as well as animals remitted from other clinics and hospitals of Valencia, was intensively sampled. This VC exclusively attended to exotic animals, not other domestic animals.

The animal study underwent review and received approval from the Animal Ethics Committee at UCH-CEU University (code of research CEEA 22/04).

### 2.2. Epidemiological Data Collection

In order to collect epidemiological information about the sampled animals, a questionnaire for each animal was filled out by the veterinarians in the practice. The survey was structured into three sections. The initial part focused on details concerning the animals’ origin and incorporated the signed informed consent from the owners. The second part covered general aspects of the animals, including their sex, age and cohabitation with other animals in the household. The concluding section encompassed clinical data, specifically addressing if the animal presented any chronic diseases and if it was under any daily medication, and lastly, inquired about its last antibiotic treatment and the specific antibiotics it has been administered throughout its life. The questionnaire is available as Supplementary Materials (Part SA).

### 2.3. Sample Collection

Between January and June 2023, samples from any small exotic mammal attending the VC were collected. A rectal swab was collected from asymptomatic animals by inserting a sterile cotton swab (Cary Blair sterile transport swabs, DELTALAB, Barcelona, Spain) into the rectum to a depth of approximately 3 cm. Subsequently, all samples were transported under refrigeration at ≤4 °C to the microbiology laboratory of the Faculty of Veterinary Sciences of the University CEU Cardenal Herrera. Microbiological analyses were performed within 24 h of sampling.

### 2.4. Salmonella Isolation

For *Salmonella* isolation, processing of all samples followed ISO 6579-1:2017 guidelines (Annex D) for the detection of *Salmonella* spp. Initially, samples underwent pre-enrichment in buffered peptone water (BPW; Scharlau^®^, Barcelona, Spain) at a ratio of 1:10 vol/vol, incubated at 37 ± 1 °C for 18 h. Subsequently, the pre-enriched samples were inoculated onto a Modified Rappaport Vassiliadis agar plate (MSRV; bioMerieux, Marcy l’Etoile, France), incubated at 41.5 ± 1 °C for 48 h. The positive MRSV plates were then transferred to two specific agar plates for the detection of *Salmonella* spp.: xylose lysine deoxycholate (XLD, Scharlau^®^, Barcelona, Spain) and a selective chromogenic agar (ASAP; bioMerieux^®^, Marcy l’Étoile, France). Both plates were incubated at 37 ± 1 °C for 24–48 h. To confirm the results, a biochemical test (API-20E, bioMerieux, Marcy l’Etoile, France) was performed. Finally, *Salmonella* isolates were serotyped using the Kauffman–White scheme [16] at the National Reference Laboratory for Animal Health (Algete, Madrid, Spain) and stored at −80 °C for further analysis.

### 2.5. E. coli Isolation

All the pre-enriched rectal swabs in BPW (Scharlau^®^, Barcelona, Spain) at a ratio of 1:10 vol/vol, were seeded on Tryptone Bile X-glucuronide agar (TBX; Scharlau, Barcelona, Spain) and incubated at 37 ± 1 °C for 24 h. Then, blue colonies showing morphology consistent with *E. coli* were picked and sown onto nutrient agar plates (Scharlau, Barcelona, Spain), followed by incubation at 37 °C ± 1 °C for 24 h. To validate the results, biochemical tests were performed (API-20E test, bioMerieux, Marcy l’Etoile, France). Finally, *E. coli* strains were kept at −80 °C for further studies.

### 2.6. Antimicrobial Susceptibility Testing

For the antimicrobial susceptibility test, two different panels of antibiotics were used with antibiotics of importance in public health. 

The first panel, carried out with the Sensititre Plate for Gram-negative bacteria EUGNF (Thermo Scientific™ Sensititre™, Madrid, Spain), and represented in Table 1, included antibiotics of public health relevance and clinically important antibiotics for companion animals included in the EARS-Vet programme [12]. 

The second panel, which was performed with the EU Surveillance *Salmonella / E. coli* EUVSEC3 Sensititre Plate (Thermo Scientific™ Sensititre™, Madrid, Spain), represented in Table 2, included the antibiotics with relevance in public health on the monitoring and reporting of antimicrobial resistance in zoonotic and commensal bacteria in food-producing animals set out in Decision (EU) 2020/1729 [17].

AMR was assessed by the minimum inhibition concentration (MIC) assay (Thermo Scientific™ Sensititre™ Plates, Madrid, Spain). All Sensititre plate results were analysed according to the breakpoints established by the European Committee on Antimicrobial Susceptibility Testing (EUCAST) in 2024 [18]. In addition, multidrug resistance (MDR) was characterised as the acquired resistance to a minimum of one agent within three or more antibiotic classes [19]. 

For this purpose, each bacterium was defrosted and cultured on nutrient agar, followed by incubation at 37 ± 1 °C for 24 h. After incubation, colonies were placed in 5 mL of sterile demineralised water (T3339; ThermoFisher Scientific™, Madrid, Spain). Each bacterial suspension was mixed and standardised to a density of 0.5 McFarland using a Nephelometer (ThermoFisher Scientific™, Madrid, Spain). Subsequently, 10 μL (for EUVSEC3 plate) and 30 μL (for EUGNF plate) of the suspension were placed in a vial containing 11 mL of Mueller–Hinton broth (T3462; ThermoFisher Scientific™, Madrid, Spain) and mixed. From this suspension, 50 μL of the vial contents were transferred into each well of the Sensititre plate. Then, after filling the wells with the inoculum, plate film was used to seal the wells and incubated at 37 ± 1 °C for 24 h. Manual reading of the plates was performed employing a Sensititre Vizion (Thermo Scientific™ Sensititre™ Vizion™ Digital MIC Viewing System, ThermoFisher Scientific, Madrid, Spain).

### 2.7. Statistical Analysis

A generalised linear model (GLM), employing the function probit link, assuming a binomial distribution for AMR patterns in *E. coli* among small mammals, was conducted for microbiological results. A *p*-value ≤ 0.05 was used as indicative of a statistically significant difference. Data are represented as least squares mean ± standard error of the least squares means. For the statistical analyses, the R software (version 4.3.1.) was used, with the EMMs [20], car [21] and multicompView [22] packages.

## 3. Results

### 3.1. Epidemiological Results

**Figure 1:**
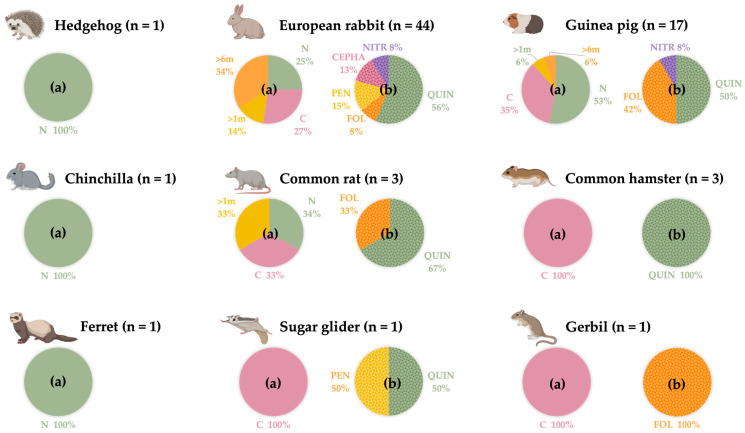
Distribution by animal species of the small mammal population studied, according to when they were last treated with antibiotics and with which antibiotic group. n: number of animals sampled. (**a**): Moment of the last antibiotic administration. N: never. C: currently. >1 m: more than a month ago. >6 m: more than 6 months ago. (**b**): Antibiotic groups administered in the study population at some point in their lives. QUIN: quinolones. FOL: folate inhibitor pathway. CEPHA: cephalosporins. PEN: penicillins. NITR: nitroimidazoles. (Created by Biorender).

In this study, a total of 72 small mammals of nine different species (Figure 1) were sampled. The studied population was divided into groups according to sex, where 52.8% (38/72) were females and 47.2% (34/72) were males, and age, where the studied population ranged from 1.5 months to 9 years. Regarding their habitat, 61.1% (44/72) of these small mammals cohabited in their households with other animals, but none of them (72/72) went out of their house. However, as the study population consists of several species from different families, these data are not directly comparable.

Regarding the clinical data collected from all the animals sampled, 25% (18/72) of them presented a chronic disease. Furthermore, with regard to the daily medication taken by the animals, 15.3% (11/72) were on some kind of medication. Finally, of all animals sampled, 68.1% (49/72) had undergone antibiotic treatment at some point in their lives, compared to 31.9% (22/72) who had never received antibiotics. The information in Figure 1 illustrates the antibiotic treatment history of each animal, including the specific antibiotic group and the date of the last treatment.

### 3.2. Salmonella and E. coli Prevalence

From the 72 specimens sampled (Table 3), 1 (1.4%) *Salmonella enterica* serovar Telelkebir 13,23:d:e,n,z_15_ strain was recovered from 1 *Oryctolagus cuniculus* (European rabbit), and 20 (27.8%) *E. coli* strains were isolated from 6 different species (Table 3).

### 3.3. Antimicrobial Susceptibility from E. coli and Salmonella Strains

Of all strains studied, 85% (17/20) of the *E. coli* isolates demonstrated resistance to at least one of the twenty-eight antibiotics tested, of which 82.6% (14/20) were MDR.

The highest frequency of AMR was observed in the quinolones group: nalidixic acid (NAL; 85%, 17/20); ciprofloxacin (CIP; 80%, 16/20); and levofloxacin (LEVO; 75%, 15/20) (*p*-value ≤ 0.05), followed by two penicillins: ticarcillin, TIC (65%, 13/20) and ampicillin (AMP; 60%, 12/20), along with sulfamethoxazole (SME; 60%, 12/20) (*p*-value ≤ 0.05). In addition, no resistance was found to nitrofurantoin (NIT), meropenem (MER) and tigecycline (TIG) (Table 4). Regarding *Salmonella*, the strain isolated was susceptible to all antibiotics tested. 

Overall, for *E. coli* isolates, 15 different AMR patterns, grouped by antibiotic class, were found. However, only two patterns were repeated, the resistance shown to the quinolones group alone, and the resistance shown to the combination of quinolones, folate inhibitor pathway, aminoglycosides and penicillins groups (Table 5).

## 4. Discussion

The present study addresses the importance of the pathogenic zoonotic bacterium *Salmonella* in small mammals, as this bacterium has been widely associated with other NTCAs, such in the case of reptile-associated salmonellosis (RAS), of which many cases have been reported worldwide [23,24,25,26]. The presented results showed a low prevalence (1.4%) of this bacterium, in accordance with a study conducted by Kylie et al., (2017) where no *Salmonella* strains were found in pet rabbits, but only in food-producing rabbits [27]. In addition, other authors have found a higher rate of these bacteria in rabbits with diarrhoeal disease, up to 30%, which may indicate that the health status of the animals may favour the colonisation of this pathogenic bacterium [28]. Regarding the rest of the animals sampled in this study, no *Salmonella* strains have been isolated. However, other studies have reported zoonotic cases in humans caused by this bacterium, isolated from hedgehogs [29], ferrets and sugar gliders [30], or Guinea pigs [31]. In this study, the results were very different from those previously obtained in reptiles when searching for this bacterium in small mammals, as only a single isolate belonging to serov. Telelkebir 13,23:d:e,n,z_15_ was found. To the authors’ knowledge, this is the first time this serovar has been described in *Oryctolagus cuniculus*. However, this serovar has been reported in human cases causing pathology worldwide, especially in cases of RAS, such as that detected in an infant from a chameleon [32]. Moreover, a total of 339 and 42 acquired cases of salmonellosis were reported between 2006 and 2016 and 2014 and 2016 by the Centres for Disease Control and Prevention (CDC) [33] and the EFSA [34], respectively, caused by this serovar. Regarding the AMR observed in this bacterium in this study, no AMR was found in this isolate. Nevertheless, other authors have detected AMR genes carried by this serovar [35], so it could pose a risk in the dissemination of these genes into the environment [36]. However, when comparing these results with those obtained from the commensal bacteria study, very different results were observed. 

In this study, *E. coli* was used, as it is considered a sentinel due to its ability to harbour AMR genes and transmit them to other commensal or pathogenic bacteria [37]. Regarding its prevalence, although more strains of *E. coli* than *Salmonella* have been isolated in the sampled species, this prevalence (27.8%) is not as high as that observed in traditional companion animals, such as dogs and cats, where the normal prevalence is around 80% [38]. Although this is not the expected result, as this commensal bacterium is present in the digestive tract of all animals, mainly mammals, it seems like a normal result, as similar prevalence has been found in other studies conducted in Canada [27] and the USA [39].

The results showed 85% AMR and 82.6% MDR, in line with other studies carried out in other Spanish regions [14,15]. These percentages are alarming, as the population of small exotic mammals is continuously growing, and in close contact with their owners, thus presenting a new challenge for authorities in the legislation of new regulations to monitor AMR and MDR in this population.

In order to control the problematic AMR and MDR represent for public health, the WHO and the European Medicines Agency (EMA) implemented a classification system for antibiotics used in animals to promote their responsible use, safeguarding both animal and public health. However, this categorisation has been updated to further control the use of antibiotics. The current WHO categorisation, published in its 7th report this year 2023 [40], includes the following categories: important antimicrobials (IAs), highly important antimicrobials (HIAs), critically important antimicrobials (CIAs), highest priority critical important antimicrobials (HPCIA) and, lastly, antibiotics not authorised for animal use, only for human medicine use.

Among the antibiotics investigated, four of them belong to the category of not authorised for animal use. They are exclusively intended for human use due to their broad-spectrum action, used in the treatment of severe infections caused by MDR bacteria in human medicine. These antibiotics, commonly recognised as last-resort antibiotics, include piperacillin/tazobactam (PIT), meropenem (MER), ertapenem (ERT) and tigecycline (TIG) [40]. Consequently, the presence of AMR is not anticipated in animals. However, this study has revealed elevated levels of AMR to ERT, consistent with findings in humans [41,42] and other animal species [43,44]. 

The highest AMR observed for *E. coli* was against the quinolones group. This could be the expected result as it was the most administered antibiotic in the study population and is the group most commonly applied in general in exotic animals [45]. However, this antibiotic group belongs to HPCIAs [40] and should therefore be restricted for use in veterinary medicine. These results stand out in comparison to those observed in conventional companion animals (dogs and cats), as for them, the main group used and with higher AMR is the penicillins group [46,47]. However, in the case of NTCAs, penicillins ranked fourth in terms of the highest AMR in this study, where AMR should practically not be observed against this group of antibiotics, as they have been taken by only a small part of the study population and are contraindicated for oral administration in lagomorphs and rodents due to their high impact on the caecal bacterial population, leading to severe intestinal dysbiosis [48,49]. 

Apart from penicillins, other groups within the class of β-lactam antibiotics have been studied, including cephalosporins and carbapenemases, previously mentioned. Regarding cephalosporins, moderate resistances have been observed. In general, this antibiotic class, as well as the aminoglycosides class, presents good effectiveness against *E. coli* infections and is one of the most common options [50]. However, none of the animals in the study population were treated with aminoglycosides and only a few with cephalosporins, so one hypothesis to explain these results could be that AMR could have been acquired through the environment, as one of the key mechanisms for the acquisition of AMR is horizontal gene transfer, which involves the transfer of AMR genes between bacteria co-inhabiting the same environment, via plasmids, conjugative transposons and other mobile genetic elements [51,52].

Similar AMR was observed for amphenicols, polymyxins and macrolides. Within these groups, the antibiotic colistin (COL) is noteworthy, since it also belongs to HPCIAs [40] and is one of the main antibiotics used to treat complicated colibacillosis infections. In the past, COL has been overused in food-producing animals, which has led to very high AMR [53] and, as a consequence, restrictions on its use and control programmes have been applied. For example, in Spain, the National Plan against Antibiotic Resistance (PRAN) [54] established the Colistin Reduce programme, which has served as an example worldwide, as it has reduced the use of this antibiotic in swine production by almost 100% [55]. Therefore, AMR to this antibiotic has decreased considerably in recent years and cases are now not so frequent, as seen in this study and in some others [53,56]. Lastly, no AMR was found in the nitrofurans group. This may be because the action mechanism is poorly understood, and it has been found to reach therapeutically effective levels only in urine [57]. Therefore, the expected results are observed in this study, as this antibiotic is underused in human and veterinary medicine.

This study presented some limitations. First, the restriction in the total sample size, which was due to the time constraint set in this study. This may affect the generalisability of the results to a wider population. Second, the difference in sample size of the different species, as for some species only a single specimen could be sampled. And finally, the detection of *Salmonella* with only one sample per animal, as the excretion of this bacterium into the environment is intermittent. However, despite these limitations, the rigorous methodology used and the consistency in data collection strengthen the reliability of our conclusions. Therefore, these promising results highlight the importance of extending this study in the future with larger sample sizes. This will help establish whether these animals are a source of *Salmonella* infection and will contribute to a better understanding of the AMR epidemiological situation in the entire Valencia region.

## 5. Conclusion

The results obtained in this study highlight the potential risk posed by the growing population of small exotic mammals in the dissemination of AMR and MDR in the environment. Consequently, there is a potential risk associated with the possession of these animals. However, these results also showed that small exotic mammals do not appear to be a source of *Salmonella*, although more large-scale studies are needed to demonstrate whether or not they really pose a threat to the spread of this bacterium in the environment. This study serves as a starting point for future plans to help control and prevent the spread of AMR and MDR. However, further studies are needed to validate our results in a larger study with more samples.

## Figures and Tables

**Table 1:** Antibiotics and their concentrations in the Sensititre Plate for Gram-negative EUGNF, and the classification of the antibiotics by the World Health Organisation.

Antibiotic Group	Antibiotic	Abbreviation	WHO	Concentration
**Aminoglycosides**	Amikacin	AMI	CIA	2–32 μg/mL
	Gentamicin	GEN	CIA	0.5–8 μg/mL
	Tobramycin	TOB	CIA	0.5–8 μg/mL
**Carbapenemases**	Ertapenem	ERT	NA	0.12–2 μg/mL
	Meropenem	MER	NA	0.12–16 μg/mL
**Cephalosporins**	Cefepime	CEP	HPCIA	0.5–8 μg/mL
	Cefixime	CIX	HPCIA	0.5–2 μg/mL
	Cefotaxime	CTA	HIA	0.5–4 μg/mL
	Cefoxitin	CXI	HIA	2–16 μg/mL
	Cefuroxime	CUR	HIA	2–16 μg/mL
	Cefalexin	CLE	HPCIA	8–32 μg/mL
	Ceftazidime	CTZ	HPCIA	0.5–8 μg/mL
**Nitrofurans**	Nitrofurantoin	NIT	NA	32–64 μg/mL
**Penicillins**	Ampicillin	AMP	HIA	2–16 μg/mL
	Amoxicillin/ Clavulanic acid	AMC	HIA	2/2–32/2 μg/mL
	Piperacillin/ Tazobactam	PIT	NA	2/4–32/4 μg/mL
	Ticarcillin	TIC	HIA	4–32 μg/mL
**Quinolones**	Ciprofloxacin (FQ)	CIP	HPCIA	0.12–1 μg/mL
	Levofloxacin (FQ)	LEVO	HPCIA	0.25–2 μg/mL
	Nalidixic acid (Q)	NAL	HPCIA	16 μg/mL
**Folate inhibitor pathway**	Sulfamethoxazole/ Trimethoprim	TRS	HIA	1/19–8/152 μg/mL
**Glycylcycline**	Tigecycline	TIG	NA	0.5–4 μg/mL

FQ: fluoroquinolone. Q: quinolone. WHO: World Health Organisation. HIA: highly important antimicrobial. CIA: critically important antimicrobial. HPCIA: highest priority critical important antimicrobial. NA: not authorised for animal use.

**Table 2:** Antibiotics and their concentrations of EU Surveillance *Salmonella / E. coli* EUVSEC3 Sensititre Plate (Thermo Scientific™ Sensititre™, Madrid, Spain), and the classification of the antibiotics by the World Health Organisation. These antibiotics are of public health importance, as set out in Decision (EU) 2020/1729.

Antibiotic Group	Antibiotic	Abbreviation	WHO	Concentration
**Aminoglycosides**	Amikacin	AMI	CIA	2–32 μg/mL
	Gentamicin	GEN	CIA	0.5–8 μg/mL
**Amphenicols**	Chloramphenicol	CHL	HIA	8–64 μg/mL
**Carbapenemases**	Meropenem	MER	NA	0.12–2 μg/mL
**Cephalosporins**	Cefotaxime	CTA	HPCIA	0.5–8 μg/mL
	Ceftazidime	CTZ	HPCIA	0.5–8 μg/mL
**Folate inhibitor** **pathway**	Sulfamethoxazole	SME	HIA	1/19–8/152 μg/mL
	Trimethoprim	TRI	HIA	0.5–16 μg/mL
**Glycylcycline**	Tigecycline	TIG	NA	0.5–4 μg/mL
**Macrolides**	Azithromycin	AZI	CIA	2–64 μg/mL
**Penicillins**	Ampicillin	AMP	HIA	2–16 μg/mL
**Polymyxins**	Colistin	COL	HPCIA	1–16 μg/mL
**Quinolones**	Ciprofloxacin (FQ)	CIP	HPCIA	0.12–1 μg/mL
	Nalidixic acid (Q)	NAL	HPCIA	16 μg/mL
**Tetracyclines**	Tetracycline	TET	HIA	2–32 μg/mL

FQ: fluoroquinolone. Q: quinolone. WHO: World Health Organisation. HIA: highly important antimicrobial. CIA: critically important antimicrobial. HPCIA: highest priority critical important antimicrobial. NA: not authorised for animal use. EMA: European Medicines Agency. A: avoid (by EMA categorisation). B: restrict (by EMA categorisation). C: caution (by EMA categorisation). D: prudence (EMA categorisation).

**Table 3:** Percentage of *Escherichia coli* isolated within each small exotic mammal species sampled.

Species	N_T_	N and (%) of *E. coli*/Animal Species
*Oryctolagus cuniculus* (European rabbit)	44	10/44 (22.7)
*Cavia porcellus* (Guinea pig)	17	5/17 (29.4)
*Rattus* norvegicus (Common rat)	3	2/3 (66.7)
*Cricetinae* (Common hamster)	3	0/3 (0)
*Chinchilla laniguera* (Chinchilla)	1	0/3 (0)
*Erinaceinae* (Hedgehog)	1	1/1 (100)
*Gerbillinae* (Gerbil)	1	0/1 (0)
*Mustela putorius furo* (Ferret)	1	1/1 (100)
*Petaurus breviceps* (Sugar gliders)	1	1/1 (100)
**Total**	**72**	**20/72 (27.8)**

N_T_: Total number of individual samples from each species. %: Percentage of each bacterium isolated from each animal species.

**Table 4:** Antimicrobial resistance in commensal *E. coli* strains isolated from small exotic mammals, per antibiotic group and per each antibiotic tested.

Antibiotic Group	% AMR of *E. coli* Isolated/Group	Antibiotic	% AMR of *E. coli* Isolated/Antibiotic
**Aminoglycosides**	36.7 ^a,b^ ± 6.2	Amikacin	25 ^a,b,c^ ± 9.7
		Gentamicin	45 ^c,e^ ± 11.1
		Tobramycin	40 ^a,c,e^ ± 11
**Carbapenemases**	15 ^c^ ± 5.6	Ertapenem	30 ^a,c^ ± 10.2
		Meropenem	0 ^g^ ± 0
**Cephalosporins**	34.3 ^a,b^ ± 4	Cefepime	30 ^a,c^ ± 10.2
		Cefixime	45 ^c,e^ ± 11.1
		Cefotaxime	30 ^a,c^ ± 10.2
		Cefoxitin	30 ^a,c^ ± 10.2
		Cefuroxime	40 ^a,c,e^ ± 11
		Cefalexin	25 ^a,b,c^ ± 9.7
		Ceftazidime	40 ^a,c,e^ ± 11
**Nitrofurans**	0 ^d^ ± 0	Nitrofurantoin	0 ^g^ ± 0
**Penicillins**	38.8 ^a,b^ ± 5.4	Ampicillin	60 ^d,e,f^ ± 11
		Amoxicillin/ Clavulanic acid	25 ^a,b,c^ ± 9.7
		Piperacillin/ Tazobactam	5 ^b,g^ ± 4.9
		Ticarcillin	65 ^d,e,f^ ± 10.7
**Quinolones**	80 ^e^ ± 5.2	Ciprofloxacin (FQ)	80 ^d^ ± 9
		Levofloxacin (FQ)	75 ^d,f^ ± 9.7
		Nalidixic acid (Q)	85 ^d^ ± 8
**Folate inhibitor pathway**	48.3 ^b^ ± 6.5	Sulfamethoxazole	60 ^d,e,f^ ± 11
		Trimethoprim	40 ^a,c,e^ ± 11
		Sulfamethoxazole/ Trimethoprim	45 ^c,e^ ± 11.1
**Glycylcycline**	0 ^d^ ± 0	Tigecycline	0 ^g^ ± 0
**Polymyxins**	25 ^a,c^ ± 9.7	Colistin	25 ^a,b,c^ ± 9.7
**Tetracyclines**	50 ^a,b^ ± 11.2	Tetracycline	50 ^c,e,f^ ± 11.2
**Amphenicols**	25 ^a,c^ ± 9.7	Chloramphenicol	25 ^a,b,c^ ± 9.7
**Macrolides**	15 ^c,d^ ± 8	Azithromycin	15 ^a,b,g^ ± 8

% AMR: antimicrobial resistance percentage (per group and per antibiotic). FQ: fluoroquinolone. Q: quinolone. ^a–g^: Distinct superscripts within each column denote statistically significant differences (*p*-value ≤ 0.05) in the resistances observed against the antibiotics examined. ±: Standard error.

**Table 5:** Number of commensal *E. coli* strains isolated from small exotic mammals resistant to different antimicrobials and their antimicrobial resistance patterns.

N of AB Groups	N of Isolates (%)	AMR Patterns
**0**	3	-
**1**	2	QUIN
**2**	0	-
**3**	1	QUIN-FOL-PEN
	1	QUIN-CEPHA-AMI
	1	QUIN-TET-POLIM
**4**	2	QUIN-FOL-AMI-PEN
	1	QUIN-FOL-PEN-TET
**5**	0	-
**6**	1	QUIN-FOL-AMI-PEN-TET-AMPH
	1	QUIN-FOL-AMI-PEN-CEPHA-TET
	1	QUIN-FOL-PEN-CEPHA-TET-AMPH
**7**	1	QUIN-FOL-AMI-PEN-CEPHA-POLIM-CARB
**8**	1	QUIN-FOL-AMI-PEN-CEPHA-TET-AMPH-CARB
	1	QUIN-FOL-AMI-PEN-CEPHA-TET-POLIM-CARB
	1	QUIN-FOL-CEPHA-TET-AMPH-POLIM-MACR-CARB
**9**	1	QUIN-FOL-AMI-PEN-CEPHA-TET-AMPH-MACR-CARB
	1	QUIN-FOL-AMI-PEN-CEPHA-TET-POLIM-MACR-CARB
**TOTAL**	**20**	

AB: antibiotic. QUIN: quinolones. FOL: folate inhibitors pathways. AMI: aminoglycosides. PEN: penicillins. CEPHA: cephalosporins. TET: tetracyclines. AMPH: amphenicols. POLIM: polymyxins. MACR: macrolides. CARB: carbapenemases.

## Data Availability

Data are contained within the article and Supplementary Materials.

## Supplementary Materials

The following supporting information can be downloaded at: https://www.mdpi.com/article/10.3390/life14020170/s1, Part SA: Questionnaire: AMR study in small mammal companion animals.
